# An Internalin A Probe-Based Genosensor for *Listeria monocytogenes* Detection and Differentiation

**DOI:** 10.1155/2013/640163

**Published:** 2013-03-20

**Authors:** Laura Bifulco, Angela Ingianni, Raffaello Pompei

**Affiliations:** Department of Biomedical Sciences, Section of Microbiology, University of Cagliari, via Porcell 4, 09124 Cagliari, Italy

## Abstract

Internalin A (InlA), a protein required for *Listeria monocytogenes* virulence, is encoded by the *inl*A gene, which is only found in pathogenic strains of this genus. One of the best ways to detect and confirm the pathogenicity of the strain is the detection of one of the virulence factors produced by the microorganism. This paper focuses on the design of an electrochemical genosensor used to detect the *inl*A gene in *Listeria* strains without labelling the target DNA. The electrochemical sensor was obtained by immobilising an *inl*A gene probe (single-stranded oligonucleotide) on the surfaces of screen-printed gold electrodes (Au-SPEs) by means of a mercaptan-activated self-assembled monolayer (SAM). The hybridisation reaction occurring on the electrode surface was electrochemically transduced by differential pulse voltammetry (DPV) using methylene blue (MB) as an indicator. The covalently immobilised single-stranded DNA was able to selectively hybridise to its complementary DNA sequences in solution to form double-stranded DNA on the gold surface. A significant decrease of the peak current of the voltammogram (DPV) upon hybridisation of immobilised ssDNA was recorded. Whole DNA samples of *L. monocytogenes* strains could be discriminated from other nonpathogenic *Listeria* species DNA with the *inl*A gene DNA probe genosensor.

## 1. Introduction

*Listeria monocytogenes* is a Gram-positive, aerobic, rod-shaped, foodborne pathogenic bacterium inducing listeriosis, an illness characterized by encephalitis, septicaemia, and meningitis [1–4]. It is the only pathogenic species of *Listeria* in humans and has been the cause of several well-documented food poisoning outbreaks [1, 5–9]. It can also cause gastroenteritis in otherwise healthy individuals and more severe invasive diseases in immunocompromised patients, pregnant women, newborns, and elderly people [10–15].

*L. monocytogenes* enters mammalian cells by inducing its own phagocytosis. Internalin A (*Inl*A) is an 80 kDa surface protein which allows *Listeria* to enter the cells. It is a complex key virulence factor protein encoded by the *inl*A gene and is specific only for *L. monocytogenes* and not for other listerial species or for other genera. It mediates the attachment of *Listeria* to, and the invasion of, hepatocytes, epithelial, and endothelial cells. The bacterial adhesion and invasion of human intestinal epithelial cells is also mediated through specific interaction with its host cell receptor E-cadherin [16–19]. 

Conventionally, the detection and identification of bacteria mainly rely on specific microbiological and biochemical identification methods, which require at least 3 and as many as 7 days to yield results. Genetic characterisation methods are more rapid than the classical identification methods and lead to unequivocal species identification [20, 21]. Among these, polymerase chain reaction (PCR), followed by hybridisation of the PCR amplified target with a labelled single-stranded oligonucleotide probe is an effective method of sequence-specific DNA detection [16, 17].

Rapid and reliable detection methods of this pathogenic, toxin-producing bacterium are required since it is able to survive and grow at low temperatures [22] and because the mortality rate for infected individuals is much higher than for other common foodborne pathogens [23–27]. 

Although there are many DNA hybridisation assays currently suitable for diagnosis, faster, cheaper, miniaturised, multianalyte, easier to use, and more sensitive approaches are highly desirable, especially in the case of decentralised analysis. In this context, electrochemical detection of DNA hybridisation events offer innovative routes [28–37].

An effective and sensitive biosensor requires a probe that can be immobilized on a sensing platform. An ideal probe should be able to achieve sensitive and specific detection of the target analyte. It must also be easy to produce and withstand environmental stresses, such as changes in temperature and pH. The proposed methodology aims at the detection of *L. monocytogenes* incidence in either environmental or clinical samples, based on the detection of the inlA gene in DNA extracts from isolated strains. For achieving our goal, we have embedded an inlA-specific probe on the surface of a SPE using several signal enhancing protocols and measured hybridization events in DNA extracts and control templates, based on generated electrochemical signals [29–31, 38–41].

At the best of our knowledge, this is the only genosensor so far described that allows the discrimination of *L. monocytogenes* from different nonpathogenic *Listeria* strains based on the detection of a specific listerial pathogenic factor such as internalin A. It proved to allow a definite and significant differentiation of pathogenic from nonpathogenic listerial species.

## 2. Materials and Methods

### 2.1. Apparatus, Chemicals, and Probe

Differential pulse voltammetry (DPV), for measurements, and cyclic voltammetry (CV), for electrode cleaning, were carried out using an AUTOLAB PGSTAT 30 electrochemical analysis system and a GPES 4.8 software package (Eco Chemie, The Netherlands). Electrodes: screen-printed gold electrodes (Au-SPEs) were obtained from Ecobioservices & Researches s.r.l. (Florence, Italy). The two 59-base oligonucleotide sequences, the *L. monocytogenes* internalin *inl*A gene probe (A) (Gen Bank M67471.1) originally designed by Ingianni et al. [16], and its complementary sequence target (B) were obtained from Invitrogen.

The sequences were as follows [16]:

 
*DNA probe* (59-base sequence A):
 5_-CCATTAGCTAATTTAACAACACTAGAACGACTAGATATTTCAAGTAATAAGGTGTCAGA-3_;
 
*DNA target* (59-base sequence B):
 5_-TCTGACACCTTATTACTTGAAATATCTAGTCGTTCTAGTGTTGTTAAATTAGCTAATGG-3_.

#### 2.1.1. Reagents, Buffers, and Solutions

Methylene blue (MB) was purchased from Difco. The Methylene blue solution was prepared with 20 *μ*M MB and 20 mM NaCl in 20 mM Tris-HCl buffer (pH 7.00). 3-mercaptopropionic acid (MPA), *N*-hydroxysulfosuccinimide (NHS), and *N*′-ethylcarbodiimide hydrochloride (EDC) were obtained from Sigma-Aldrich (Steinheim, Germany). All chemicals were of an analytical reagent grade. In-house distilled and sterilised water was used for the preparation of all buffers and solutions. 50 mM H_2_SO_4_ solution was used for electrochemical cleaning of the electrodes.

#### 2.1.2. Microbial Strains and Conditions

6 listerial strains from foods (Lys 1: *L. innocua*, Lys 2: *L. monocytogenes*, Lys 3: *L. monocytogenes*, Lys 4: *L. monocytogenes*, Lys 5: *L. monocytogenes*, and Lys 6: *L. ivanovii*) and 4 listerial collection strains (Lys 7: C 315 *L. innocua*, Lys 8: C 276 *L. innocua*, Lys 9: C 383 *L. monocytogenes*, and Lys 10: C 483 *L. monocytogenes*) taken from our Institute's collection and representing important species of the genus *Listeria* were used in this work. DNA samples were prepared as described. The strains were grown on BHI plates and reidentified by metabolic tests according to Ingianni et al. [16] and use of the API *Listeria* galleries (bioMèrieux Italia, Milan, Italy). 

#### 2.1.3. DNA Extracts

DNA samples were prepared as follows: the bacteria strains were incubated overnight in BHI broth and washed twice in PBS before DNA extraction using an Easy-DNA kit (Invitrogen, Carlsbad, Ca, USA) following the manufacturer's protocol. DNA concentration and purity were determined by UV light absorbance measured by an Ultrospec III spectrophotometer (Pharmacia LKB). 

#### 2.1.4. PCR Performance

PCR was performed as described by Ingianni et al. [16]. DNA oligonucleotide stock solutions (100 mg/L) and *Listeria* DNA extracts (dsDNA 100 mg/L) were prepared with TE solution (10 mM Tris-HCl, 1 mM EDTA, and pH 8.00) and kept frozen. Working DNA solutions were prepared with either 500 mM acetate buffer (pH 4.80) or 20 mM Tris-HCl buffer (pH 7.00), according to the hybridisation protocol [38]. 

### 2.2. SAM Preparation and Electrode Modification

The SAM modification of Au-SPEs was performed following a protocol described by Gooding et al. [38] and Kerman et al. [39] for gold rod electrodes. This protocol was adjusted for screen-printed electrode modification. The gold surfaces of the working electrodes were prepared by electrochemical cleaning before modification. The electrodes were cleaned by cycling between the 0 V and +1.5 V potentials in a 50 mM H_2_SO_4_ solution at a scan rate of 100 mV/s for approximately 15 min. until reproducible scans were recorded. The electrodes were rinsed with sterile distilled water before SAM modification.

SAMs were prepared by covering the surfaces of the clean Au-SPEs with a freshly prepared 75 : 25 (v/v) ethanol:water solution containing 20 mM MPA. Au-SPEs were incubated in this ethanolic solution overnight for approximately 15 h. The Au-SPEs/SAM were rinsed with 75 : 25 (v/v) ethanol:water and then with water, prior to covalent activation by immersion in the 50 mM phosphate buffer solution (pH 7.40) containing 2 mM EDC and 5 mM NHS for 1 h. Then, the Au-SPEs/SAM/Linker surfaces were rinsed with the 50 mM phosphate buffer solution (pH 7.40). 

Next, DNA immobilisation was performed on the working electrode surfaces. 20 *μ*L of 500 mM acetate buffer solution (pH 4.80) containing 100 ppm probe were pipetted onto the surface of each Au-SPEs/SAM/Linker. The probe droplets were left to air-dry overnight. Sensors were then soaked in water for 2 h and rinsed again with water to remove unbound DNA. Thus, *inl*A probe-modified Au-SPEs were obtained. Three *inl*A probe-modified electrodes were utilised as a control (*inl*A probe) for each experiment. The cost of each electrode was about 1.5 euros, and it was found to be stable for at least 1 week, when kept in the refrigerator.

### 2.3. Hybridisation

20 *μ*l of 20 mM Tris buffer solution (pH 7.00) containing 100 ppm target (complementary sequences or whole *Listeria* DNAs) were pipetted onto the *inl*A probe-modified Au-SPE surfaces. Whole *Listeria* DNA samples were prepared immediately before hybridisation by high temperature denaturation (94°C) for 10 min. to obtain ssDNA. The target droplets were air-dried for 30 min. This allowed hybrid-modified Au-SPEs to be obtained. Each test required about 50–60 min of work by a technician.

### 2.4. MB Binding

MB was accumulated on the surface of either the modified or the hybridised electrodes, by pipetting 20 *μ*L of 20 mM Tris-HCl buffer (pH 7.00) containing 20 mM MB with 20 mM NaCl, which was then left for 5 min. without applying any potential. After MB accumulation, the electrodes were rinsed with 20 mM Tris-HCl buffer (pH 7.00) for a few seconds.

### 2.5. Voltammetric Transduction

The reduction signal of the accumulated MB was measured by using differential pulse voltammetry (DPV) with an amplitude of 10 mV and scan rate of 20 mV/s. Experiments were carried out in 20 mM Tris-HCl buffer (pH 7.00). Each experiment was carried out in triplicate.

## 3. Results and Discussion

### 3.1. Genosensors

The genosensors relied on the electrochemical transduction of the hybridisation between the immobilised ssDNA probe and its unlabelled complementary sequences. By following the modified protocol, we could form the SAMs on the surfaces of the screen-printed gold electrodes and activate them. Then, the original *inl*A probe was covalently linked onto the gold-working electrodes. The sensors were optimised for use with the complementary oligonucleotide and then tested on samples of *Listeria* culture DNA extracts. Hybridisation detection was accomplished by measuring the MB reduction signal. Electroactivity of this label could discriminate the hybrid from the probe. The decrease in the magnitude of the MB voltammetric reduction signals, thus, reflected the extent of hybrid formation. Probe specificity and probe-method sensitivity were further tested using PCR products of *inl*A gene targets as templates.

### 3.2. Probe Immobilisation

To understand probe coverage and surface organization at the Au-SPEs/SAM, the Au-SPEs/SAM/activated, and the Au-SPEs/SAM/probe-modified electrodes, we recorded peak current magnitudes at the respective electrodes after incubation in MB solutions.

**Figure 1:**
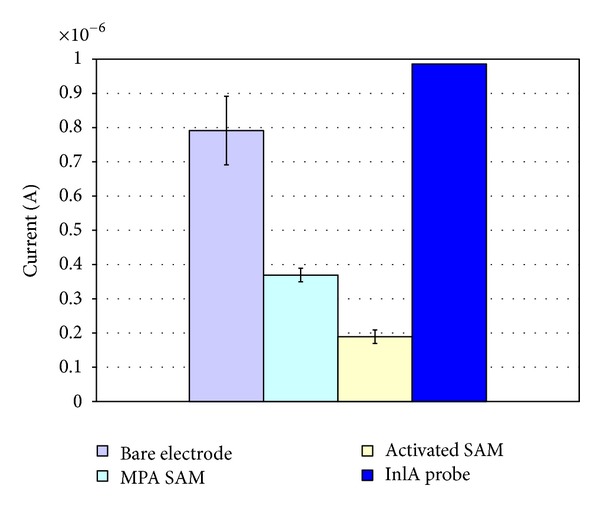
Comparison of the MB reduction peaks. MB reduction at the bare electrode (first gray column), after SAM modification (second green column) and activation (third pale yellow column) steps and after *inl*A probe covalent binding (last blue column) (average of 10 electrodes; *inl*A probe versus MPA, activated SAM, and bare electrode: *P* < 0.05).

Measurements of MB reduction were carried out at the bare electrodes (Figure 1), at the MPA-SAM modified electrodes, at the EDC/NHS-activated SAM electrodes and at the SAM/ssDNA *inl*A probe-modified electrodes. The voltammetric signal of MB reduction at the bare electrodes decreased after SAM modification and activation and increased again after *inl*A probe linking. The MPA-SAM restricted MB access to the electrode but still allowed significant electrochemistry to occur at the underlying electrode (MPA-SAM in Figure 1). The activation of this carboxylic acid terminated SAM with EDC/NHS further restricted MB access to the electrode without completely passivising it (activated SAM in Figure 1). Immobilisation of the probe on the SAM-modified electrodes resulted in an increase in MB peak currents (*inl*A probe in Figure 1) due to the affinity of MB for the free guanine bases of the DNA as previous reported [30, 42]. The values of inlA probe against bare, SAM, and activated SAM were found to be significant: *P* < 0.05.

### 3.3. Detection of the *Inl*A Complementary DNA Sequence

**Figure 2:**
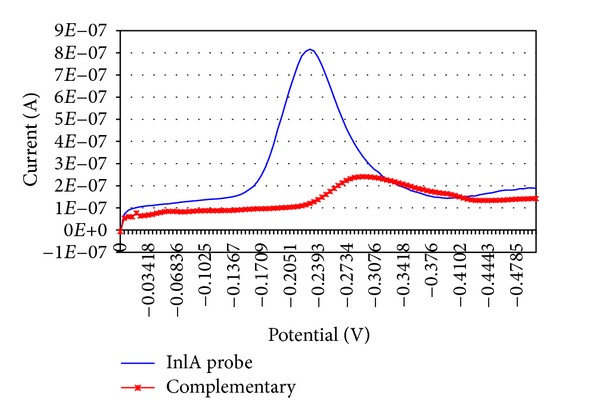
DP voltammograms of *inl*A probe-modified electrodes and hybrid-modified electrodes with the complementary sequence.

The sensors were studied for hybridisation detection using the complementary sequence of the immobilised probe. The genosensors were usable for one shot only. Therefore, we compared the data obtained from series of 3 to 5 genosensors produced during each experiment. Data shown are the average of each experiment.  Figure 2 shows the DP voltammograms for the MB reduction signal at the inlA probe-immobilised Au-SPEs (blue) and after hybridisation with the target (red). The shown voltammetric curves are the average of 5 electrodes. The highest MB reduction signal was observed with the ssDNA probe on the electrode alone (Figure 2, blue), because MB has a strong affinity for the free guanine bases; hence, the greatest amount of MB accumulation occurs on this surface. An obvious decrease in the voltammetric peak was observed for the indicator after double-strand formation (Figure 2, red), since the interaction between MB and the guanine residues of the probe was prevented by hybrid formation on the electrode surface.

**Figure 3:**
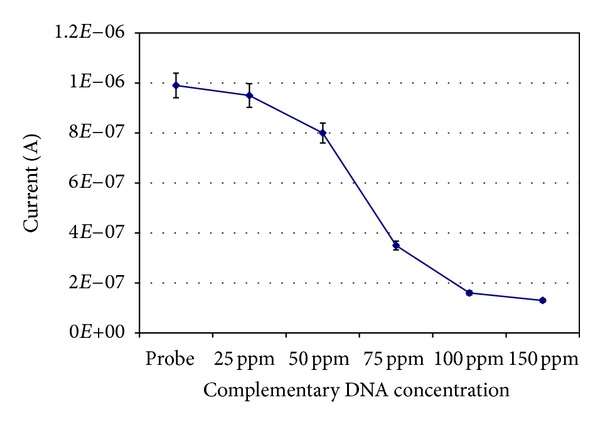
Detection of the *inl*A complementary DNA sequence. Different concentrations of complementary DNA were tested versus 100 ppm probe. Each point is the average of 5 genosensors. The MB reduction signal decreases to a plateau as the hybridisation increases.

**Figure 4:**
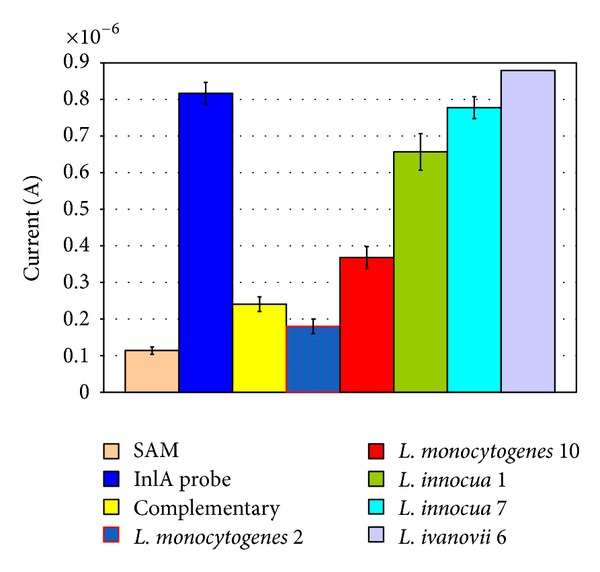
Mb reduction test of pathogenic and nonpathogenic listerial species. Comparison of two samples of *L. monocytogenes*, two samples of *L. innocua,* and one sample of *L. ivanovii* versus probe and complementary oligonucleotide (the media of *L. monocytogenes* against other *Listeria* species was statistically highly significant: *P* = 0.0016). Probe, complementary, and DNA samples concentrations were 100 ppm.

The sensors were tested with different concentrations of complementary oligonucleotide. A voltammetric signal was still observed (Figures 3 and 4), even when all the DNA probe was completely hybridised to a duplex, because MB can also act as an intercalator. However, the rapid decrease of the MB signal after hybridisation, as shown in the calibration curve, indicates that the voltammetric signal due to intercalation is small compared to the signal from direct interaction with the guanine bases.

### 3.4. Detection of *L. monocytogenes* Strains and Discrimination from Different *Listeria* Strains

Genosensors were tested on whole DNA samples of different *L. monocytogenes* strains. The differences recorded in the reduction signals indicate a different grade of hybridization between *L. monocytogenes* strain DNAs. It could be due to the presence of a different number of copies of the *inl*A gene; thus, the voltammogram peaks of *L. monocytogenes* DNAs showed a minimum high when a low number of copies are present, while *L. monocytogenes* peaks were higher for a larger number of copies of the gene.

Genosensors were then tested on whole DNA samples of different *Listeria* strains.  Figure 4 shows the comparison of MB reduction peaks after hybridisation with two DNA samples of *L. monocytogenes* strains, one from *L. ivanovii* and two DNA samples of *L. innocua* strains. The *inl*A gene sequences are only present in the DNA of *L. monocytogenes*. 

However, the *L. monocytogenes* voltammogram current was always lower than *Listeria* non-*monocytogenes* voltammograms (the difference between the media of *L. monocytogenes* and the media of other *Listeria* species was found to be highly statistically significant: *P* = 0.0016). 

**Figure 5:**
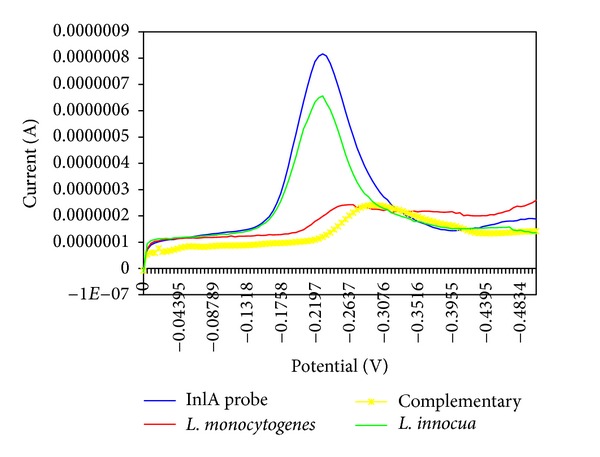
Voltammograms of listerial strains. Two hybridisation voltammograms of *L. monocytogenes* and *L. innocua* are shown in comparison with an *inl*A probe voltammogram and a hybridisation test with a complementary sequence voltammogram.

MB reduction peak data of the six *L. monocytogenes* strains were compared with the voltammograms of the other strains.  Figure 5 shows that the *L. innocua* voltammograms are comparable to the inlA probe signals, while *L. monocytogenes* voltammograms are comparable to the complementary oligonucleotide signals.

Experiments were carried out to investigate genosensor stability. They were stored at 4°C after preparation, and measurements were performed after 24, 48, 72, and 96 hours. When kept in a freezer at −20°C the genosensors presented the same responses for at least 6 weeks, and at −80°C, they were still efficient after 3 months. 

## 4. Conclusions

In this work, we investigated the possibility of an Internalin A (*inl*A) probe application for the construction of a genosensor for the identification of the pathogenic bacterium *L. monocytogenes*. The *inl*A probe utilised was previously designed in our laboratory. Due to the formation of the alkanethiol SAMs adsorbed on Au-SPE surfaces, the *inl*A probe could be attached using covalent linkers such as EDC and NHS. These genosensors were used for the detection of hybridisation on the Au-SPE surfaces by means of MB as the electroactive reporter. The *inl*A probe-modified Au-SPEs were shown to transduce hybridisation with complementary and, more interestingly, with whole DNA samples of *L. monocytogenes* that contained the *inl*A gene. Furthermore, discrimination between different pathogenic and nonpathogenic *Listeria* species was recorded. The test is quite inexpensive, requires less than 60 min of technical work, when DNA extracts are available, and can be useful for assaying DNA extracted from clinical isolates, as well as from environmental and food strains, especially in the case of decentralised analysis.
